# *Bacillus amyloliquefaciens* CECT 5940 alone or in combination with antibiotic growth promoters improves performance in broilers under enteric pathogen challenge

**DOI:** 10.3382/ps/pez223

**Published:** 2019-04-19

**Authors:** Marllon José Karpeggiane de Oliveira, Nilva Kazue Sakomura, Juliano Cesar de Paula Dorigam, Kiran Doranalli, Letícia Soares, Gabriel da Silva Viana

**Affiliations:** 1 Department of Animal Science, Universidade Estadual Paulista “Julio de Mesquita, Filho”, FCAV/UNESP, Jaboticabal, São Paulo 14884–900, Brazil; 2 Evonik Nutrition & Care GmbH, Hanau, 63067, Hessen, Germany

**Keywords:** *Clostridium perfringens*, direct-fed microbial, *Eimeria maxima*, necrotic enteritis, probiotic

## Abstract

A study was conducted to investigate the effects of *Bacillus amyloliquefaciens* CECT 5940 as a direct-fed microbial (DFM) alone or in association with bacitracin methylene disalicylate (BMD) in broilers under enteric pathogen challenge. A total of 1,530-day-old male Cobb500 chicks were randomly assigned to 5 treatments, with 9 replicate pens with 34 birds each. Treatments included positive control (PC, basal diet without additives or challenge); negative control (NC, basal diet without additive and challenged birds); NC + 0.05 g/kg BMD; NC + 1 g/kg DFM (10^6^ CFU *B. amyloliquefaciens* CECT 5940/g of feed); and NC + 0.05 g/kg BMD + 1 g/kg DFM. The challenge consisted of oral gavage with *Eimeria maxima* and *Clostridium perfringens* inoculum. Body weight gain (BWG), feed intake (FI), and feed conversion ratio (FCR) were evaluated on days 21, 35, and 42. Ileal and cecal content were collected on days 21 and 28 for *C. perfringens* enumeration by real-time PCR assay and the intestinal health was evaluated by scores. Uniformity (UN), carcass (CY), and breast meat yields (BMY) were evaluated on day 42. After 14 and 21 d post-inoculation, birds in the challenged groups had significant lower FI and BWG compared to the PC group (*P* < 0.05). However, the groups receiving DFM, BMD, or its combination presented better FCR, CY, BMY, UN, and lower incidence of footpad lesion and litter quality visual scores, compared to the NC group without feed additives (*P* < 0.05). Mortality was not affected by treatments (*P* > 0.05). Broilers fed DFM, BMD, or its combination presented lower *C. perfringens* in ileal content at 21 and 28 d compared to NC group without additives (*P* < 0.05) and also maintained gut health by keeping the frequency of ballooning, abnormal content, and swollen mucosa comparable to the PC group (*P* > 0.05). The study indicates that *Bacillus amyloliquefaciens* CECT 5940 is effective as BMD to provide similar performance and gut health in challenged broilers.

## INTRODUCTION

*Clostridium perfringens* is a gram-positive bacteria that naturally occur in intestinal microbiota without compromising gut health (Timbermont et al., 2009; Miller et al., 2010; Prescott et al., 2016; Bhogoju et al., 2018). However, the high dietary inclusions of protein sources of animal origin such as fishmeal (Drew et al., 2004; Fernandes da Costa et al., 2013; Stanley et al., 2014) and cereals with high non-starch polysaccharides content (Annett et al., 2002; Timbermont et al., 2011; Barekatain et al., 2013; Latorre et al., 2015) or enteric coccidial infections might result in overgrowth of *C. perfringens* population as well as the increase in bacterial toxin production that may lead to necrotic enteritis (Keyburn et al., 2010; Yan et al., 2013; Zhou et al., 2017). Necrotic enteritis (**NE**) is an avian disease responsible for economic losses of approximately $6 billion (US) annually (Wade and Keyburn, 2015). Clinical signs of NE include feed consumption depression, ruffled feathers, severe necrosis of the intestinal tract, diarrhea, and a sudden rise in flock mortality, while subclinical challenge with *C. perfringens* is characterized by a less severe depression in voluntary feed intake (**FI**) and damages to the intestinal mucosa (Porter, 1998; Van Immerseel et al., 2009; Cooper et al., 2013; Du et al., 2015).

Under commercial conditions, subtherapeutic doses of antibiotics have been included in feed in order to prevent the overgrowth of potential pathogenic bacteria such as the mentioned *C. Perfringens* and also as a growth promoter (**AGP**). However, the application of subtherapeutic doses of antibiotics for a long period may increase antimicrobial resistance in pathogens of importance for animal and human health and this concern led to regional bans in order to reduce AGPs use in poultry feed (Dibner and Richards, 2005; M'Sadeq et al., 2015; Lekshmi et al., 2017). Such framework inevitably led the industry to look for valid alternatives that support adequate broiler’s health and performance like AGPs.

Probiotic, also known as direct-fed microbial (DFM), is a potential replacement for AGPs (Patterson and Burkholder, 2003; M'Sadeq et al., 2015; Clavijo and Flórez, 2018) and can also help to decrease the dietary supplementation of AGP when supplied together. Probiotics are live microorganisms which when administered in adequate amounts confer a health benefit on the host (Reid et al., 2003). *Bacillus* species have been shown to be viable probiotics, whose benefits for host includes the suppression of pathogen colonization (Wu et al., 2018), improvements on growth performance (Rathnapraba et al., 2018), nutrient digestibility (Sen et al., 2012; Hossain et al., 2015), modulation of immune responses (Paszti-Gere et al., 2012; Gadde et al., 2017), and maintenance of gut integrity (Al-Baadani et al., 2016; Jayaraman et al., 2017). *Bacillus amyloliquefaciens* CECT 5940 is a probiotic used in poultry feeds due to the positive effects on pathogenic bacteria such as *C. perfringens* or *E. coli* (Diaz, 2007; Lei et al., 2015).

Even though several research efforts have been focused on investigating the benefits of *Bacillus amyloliquefaciens* on broiler growth performance, few reports were conducted to achieve such purpose under the condition of an enteric pathogen challenge. Given this background, we hypothesized that dietary supplementation of *Bacillus amyloliquefaciens* CECT 5940 in challenged-broilers could support similar growth rates to diets containing AGP. Therefore, the purpose of the current study was to evaluate the effects of supplementation with *Bacillus amyloliquefaciens* CECT 5940 on performance, carcass traits, and gut health of broilers under enteric pathogen challenge.

## MATERIALS AND METHODS

### Ethical Committee Statement

The experimental protocol used in the current study was previously approved by the Ethics Committee of Animal Care and Use of the Universidade Estadual Paulista “Julio de Mesquita Filho”, FCAV/UNESP, Jaboticabal, São Paulo, Brazil (protocol N° 010300/17).

### Bird Husbandry and Experimental Design

A total of 1,530-day-old male Cobb500 chicks with initial body weight of 50.2 ± 0.1 g were used in this study. The study was conducted in a completely randomized design with 5 treatments and 9 pen replicates with 34 birds each. Birds were raised in pens (2.0 × 1.5 m) with the floor covered by wood shavings. All pens were equipped with nipple drinkers and tubular feeders, which provided ad libitum access to feed and water for the entire 42-D feeding trial.

### Diets and Experimental Treatments

A basal diet, composed mainly of corn and soybean meal, was formulated to meet the nutritional requirements (Rostagno et al., 2017) of broilers in the starter (day 1 to 13), grower I (day 14 to 21), grower II (day 22 to 35), and finisher (day 36 to 42) phases. From the basal diet (Table 1), 5 treatments were produced: (1) positive control (**PC**, basal diet without additives or challenge); (2) negative control (**NC**, basal diet without additive and challenged birds); (3) NC + 0.05 g/kg bacitracin methylene disalicylate (**BMD**); (4) NC + 1 g/kg DFM (1 × 10^6^ CFU *B. amyloliquefaciens* CECT 5940/g of feed); and (5) NC + 0.05 g/kg BMD + 1 g/kg DFM.

**Table 1.** Composition (%) of the basal experimental diet.

	Starter	Grower I	Grower II	Finisher
Ingredients (%)	Day 1–13	Day 14–21	Day 22–35	Day 36–42
Corn	55.53	62.71	66.10	65.84
Soybean meal (45%)	35.68	29.19	25.98	25.50
Meat and bone meal (43%)	3.00	3.00	3.00	3.00
Soybean oil	3.34	2.94	3.08	3.87
Dicalcium phosphate	1.00	0.85	0.59	0.59
Salt (NaCl)	0.47	0.47	0.47	0.47
Choline chloride (60%)	0.10	0.10	0.10	0.10
Vitamin premix^1^	0.05	0.05	0.05	0.05
Mineral premix^2^	0.05	0.05	0.05	0.05
DL-Methionine (99%)	0.32	0.26	0.23	0.23
L-Lysine (54.6%)	0.32	0.28	0.27	0.22
L-Threonine (98.5%)	0.09	0.07	0.07	0.05
L-Valine (96.5%)	0.06	0.02	0.01	0.01
Nutritional composition (%)				
Metabolizable energy, kcal/kg	3,050	3,100	3,150	3,200
Calcium	0.90	0.84	0.76	0.76
Av. phosphorus	0.58	0.54	0.48	0.48
Sodium	0.21	0.21	0.21	0.21
Potassium	0.85	0.76	0.71	0.70
Crude protein	23.68 (24.17)	21.15 (21.50)	19.90 (19.64)	19.60 (18.50)
Lysine	1.40 (1.45)	1.21 (1.27)	1.12 (1.12)	1.08 (1.04)
Methionine + cystine	1.01 (0.95)	0.90 (0.85)	0.84 (0.80)	0.83 (0.77)
Threonine	0.94 (0.92)	0.83 (0.81)	0.78 (0.74)	0.76 (0.71)
Valine	1.13 (1.12)	0.99 (0.98)	0.92 (0.89)	0.91 (0.87)
Isoleucine	0.97 (0.97)	0.85 (0.85)	0.80 (0.77)	0.78 (0.75)
Arginine	1.55 (1.57)	1.36 (1.37)	1.26 (1.22)	1.24 (1.19)
SID^3^ lysine	1.26 (1.31)	1.09 (1.14)	1.01 (1.01)	0.97 (0.93)
SID methionine + cystine	0.91 (0.86)	0.81 (0.77)	0.76 (0.72)	0.75 (0.70)
SID threonine	0.80 (0.78)	0.70 (0.68)	0.66 (0.63)	0.64 (0.60)
SID valine	1.00 (0.99)	0.87 (0.86)	0.81 (0.78)	0.80 (0.76)
SID isoleucine	0.86 (0.76)	0.76 (0.76)	0.71 (0.68)	0.70 (0.67)
SID arginine	1.41 (1.43)	1.23 (1.24)	1.14 (1.10)	1.12 (1.07)

Analyzed content in parenthesis.

^1^Provided per kg of vitamin premix: folic acid (min) 1,600 mg; vitamin B_5_–pantothenic acid (min) 24.96 g; biotin (min) 80 mg; butyl hydroxide toluene 100 mg; niacin (min) 67.20 g; selenium (min) 600 mg; vitamin A (min) 13,440,000 UI; vitamin B_1_ (min) 3,492 mg; vitamin B_12_ (min) 19,200 mcg; vitamin B_2_ (min) 9,600 mg; vitamin B_6_ (min) 4,992 mg; vitamin D_3_ (min) 3,200,000 UI; vitamin K_3_ (min) 2,880 mg.

^2^Provided per kg of mineral premix: copper (min) 15 g; iron (min) 90 g; iodine (min) 1,500 mg; manganese (min) 150 g; zinc (min) 140 g.

^3^SID, standardized ileal digestible (values in parenthesis are calculated using total AA analyzed and the SID coefficients from AMINODAT 5.0).

### Challenge Model

The enteric pathogen challenge model used in this study was designed based on a subclinical in vivo model described previously (Onrust et al., 2018) with some minor modifications. The method combines a *C. perfringens* challenge with a coccidial challenge. With the purpose of inducing the onset of a *C. perfringens* infection, a mild *Eimeria maxima* infection was used as a trigger. At 17 D, all birds in the challenged groups were individually inoculated by oral gavage with 1 mL of *Eimeria maxima* inoculum (approximately 3.85 × 10^4^ sporulated oocysts) and, afterwards, inoculated with 1 mL of broth fresh culture of *C. perfringens* (approximately 2.5 × 10^6^ CFU) per bird on days 18, 19, and 20.

The birds were inoculated with the strain of *Clostridium perfringens* ATCC 13124 (alpha-toxin producer), which was kindly provided by the National Institute of Quality Control in Health (INCQS) of the Oswaldo Cruz Foundation (Rio de Janeiro, Brazil). Briefly, the strain was grown in brain heart infusion broth on agar (Oxoid) under anaerobic conditions at 37°C for 72 h. The culture of *C. perfringens* was placed on the tubes containing brain heart infusion and incubated according to Boarini et al. (2015) and PCR was used to confirm the presence of phospholipase C (*plc* gene; GenBank accession number KY584046). Whole suspended culture was then centrifuged under 5000× *g* for 10 min at 4°C, the bacterial pellets were collected and concentered to 10^−7^ using the McFarland Standards. The bacterial count was made as described by Boarini et al. (2015) and final concentration of *C. perfringens* inoculum was 2.5 × 10^6^ CFU/mL. The inoculum of *Eimeria maxima* sporulated oocysts was obtained by a commercial laboratory (Jaboticabal, São Paulo, Brazil) at the concentration of 3.85 × 10^4^ oocyst/mL.

### Performance and Carcass Trait Measurements

Live body weight and FI were recorded at 21, 35, and 42 D for further calculation of body weight gain (**BWG**) and feed conversion ratio (**FCR**). The cause, date, and weight of dead birds were recorded daily. Mortality was used to correct FCR. All birds were weighed individually to determine flock uniformity on day 42. Three broilers, whose weight represented the average BW of each pen, were selected for carcass evaluation (total carcass yield and breast meat yield). Broilers were euthanized, bled, scalded (60°C/120 s), plucked and eviscerated. After removing neck, head, and feet, carcasses were weighed (**CW**) so that the carcass yield could be calculated (CW/live body weight). Subsequently, breast weight (**BW**) was measured to calculate breast meat yield (BW/CW).

### Foot Pad Dermatitis and Litter Quality Measurements

Foot pad dermatitis (**FPD**) was scored on the same 3 euthanized birds used for carcass trait measurements. Chickens were individually scored using the 5-point Welfare Quality recommended scale (Welfare Quality, 2009). The scores ranged from 0 (no evidence of pododermatitis) to 4 (severe pododermatitis). At day 28, litter quality was evaluated. For such, the assessment within the pen was done from the litter floor at each 4 corners and the values of each treatment represent the mean of 36 observations (4 corners × 9 replicates). Litter samples were visually scored on a scale of 0 to 5 as follows: 0 = dry friable; 1 = dry with very fine texture; 2 = sticky on compression or crumbles; 3 = clod on compression; 4 = wet; 5 = drops of water come out on compression.

### Intestinal Sample Collection and Genomic DNA Extraction

On days 21 and 28, 2 birds were randomly selected from each group, euthanized to collect ileal and cecal contents. The digesta content of ileum (from Meckel's diverticulum to the ileo-cecal junction) and caeca from 2 broilers per pen were collected aseptically, pooled, and stored in a germ-free universal collector at −80°C separately for further analysis.

The homogenate of the ileum and caeca content from each pool (a total of 9 samples per treatment) was weighed and recorded (45 ± 10 mg). Bacterial Genomic DNA was extracted directly from the pool of ileal and cecal content using commercial reagents (NewGene), following manufacture's specifications (Simbios Biotecnologia, Cachoeirinha, RS, Brazil). Briefly, each pool was mixed with 1,250 μL of lysis solution (5 M guanidine thiocyanate, 0.1 M Tris-HCl [pH 6.4]), vortexed vigorously and incubated at 60°C (±5°C) for 10 min. After centrifugation (10,000 × *g*, 1 min), 500 μL of the supernatant was transferred to a tube containing 20 μL of a silica suspension. After agitation and centrifugation (10,000 × *g*, 1 min), the pellet was rinsed with 150 μL of solution A (5 M guanidine thiocyanate, 0.1 M Tris-HCl [pH 6.4]), then with solution B (75% ethanol), and lastly with solution C (absolute ethanol). After last rinsed, the silica was dried at 60°C for 10 min and total DNA was eluted in 50 μL of EL buffer (10 mM Tris-HCl [pH 8.0], 1 mM EDTA).

### Real-Time PCR Assay

The *plc* gene was targeted using the primers and probe as described by Abildgaard et al. (2010) based on *plc* gene sequences from 60 different strains of *C. perfringens* isolated from broilers as described in Table 2. The TaqMan probe was labeled on the 5′ end with the fluorescent 6-carboxyfluorescein (FAM) and with non-fluorescent quencher dyes at the 3′ end. Bacterial populations were quantified by absolute quantitative real-time PCR. The samples were amplified by commercial NewGene CPRAmp Master Mix (Simbios Biotecnologia, Cachoeirinha, RS, Brazil). The reactions were performed in a total of 30 μL volume, with 2.0 μL of extracted DNA, 1.5 U of Taq DNA Polymerase, and final concentration of 1.5 mM MgCl_2_, 0.25 μM of each primer and 0.125 μM of the probe. All real-time Taqman PCRs were performed on StepOne Plus (Applied Biosystems), and thermocycler conditions were one cycle of 3 min initial denaturation at 95°C, and 40 cycles of denaturing at 95°C (15 s) and annealing/extension at 60°C (1 min). Data were collected at all temperature steps and analyzed using the StepOne Software v2.0.2 (Applied Biosystems). For quantification, a double-stranded DNA fragment (gBlock Gene Fragment, Integrated DNA Technologies, Iowa, USA) was designed containing target regions of alpha-toxin-encoding gene (*plc*) amplified by the primers described above and designed based in *C. perfringens* strain ATCC 13124 alpha-toxin (*plc* gene; GenBank accession number KY584046). A 10-fold serial dilution of gBlock was used to generate a standard curve between log standard concentrations (4.0 × 10^5^ to 4.0 × 10^1^ copies per reaction) and quantification cycle (**C_q_**) values. Samples that showed signals crossing the threshold line in both replica until C_q_ value of 40 and presented a characteristic sigmoid curve were regarded as positive. To limit of detection and conversion of PCR cycle threshold values to bacterial cell numbers, a standard curve was constructed using a DNA extracted from the pure culture of *C. perfringens* (ATCC 13124). The DNA was diluted using the serial dilution method from 2.41 × 10^7^ cell/mL until 2.41 × 10^0^ and subjected to the real-time PCR procedure. A plot of C_q_ vs log cell numbers was created and PCR efficiency was calculated from the slope of this graph using the equation E = 10^(–^^1/slope)^ – 1 (Rasmussen, 2001). For all amplification assay this graph of calibration curve, the y intercept, and r² were determined.

**Table 2.** Oligonucleotides used in this study.

Target	Name	Type	Sequence 5′-3′
*Clostridium perfringens* (*plc* gene)	398F	Forward primer	CTA GAT ATG AAT GGC AAA GAG GAA ACT A
	475R	Reverse primer	AAC ATT GCA GGA TGA TAT GGA GTA GTA TCT AT
	430T	TaqMan probe	CAA GCT ACA TTC TAT CTT GGA GAG GCT ATG CAC TAT TT

### Intestinal Evaluation

On days 21 and 28, 2 broilers per pen were randomly selected (18 birds/treatment) and euthanized for intestinal health analysis. The intestinal condition was analyzed according to Teirlynck et al. (2011) where it is attributed values of 1 (presence) or 0 (absence) for each one of the 7 parameters evaluated: (1) ballooning in the gut; (2) gut mucosa sloughed; (3) significant swollen or redness of the serosal or mucosal or both surface of the gut; (4) thin or fragile intestinal wall; (5) abnormal appearance of the contents in the lumen (excessive slime, water, gas, greasy aspect or mixture of these); (6) presence of undigested food particles, and (7) muscle tone.

The evaluations were represented as “frequency of occurrence” (% *F_i_*), defined as the percentage in which each specific variable occurred on all observations. It was calculated as follows: % *F_i_* = (N*_i_*/N) × 100, where: N*_i_* = number of positive observations containing variable i and N = the total number of observations.

### Statistical Analysis

Data were analyzed as a one-way ANOVA using the GLM procedure of SAS software 9.4 (SAS Institute Inc, 2013). Each pen was considered as experimental units. Means were compared using Student–Newman–Keuls multiple range test where appropriate. The evaluation of the intestinal health was analyzed by the non-parametric Kruskal–Wallis test, followed by Dunn-Bonferroni, as the data were not normally distributed. All statements of significance are based on the 0.05 level of probability.

## RESULTS

### Performance, Carcass Traits, and Uniformity

Broiler performance responses are detailed in Table 3. There were no significant differences for BWG and FCR between treatments on day 21 (*P* > 0.05). However, FI was significantly lower in the PC and DFM groups compared to NC group without feed additives (*P* < 0.05), but not significantly different from the groups receiving BMD or its combination with DFM (*P* > 0.05). On days 35 and 42, BWG and FI were significantly lower in the challenged groups receiving additives or not comparable to the PC group (*P* < 0.05). On the other hand, FCR was improved in the groups receiving DFM, BMD or its combination compared to the NC group without additives, but still significantly higher than the PC group (*P* < 0.05). As presented in Table 4, broilers fed DFM, BMD, or its combination had better uniformity, carcass, and breast meat yields compared to the NC group without feed additives (*P* < 0.05) and values similar to the PC group (*P* > 0.05).

**Table 3.** Effects of supplementation with *Bacillus amyloliquefaciens* CECT 5940 probiotic (DFM), BMD, or its combination on performance parameters of challenged broiler.

	Day 1–21			Day 1–35			Day 1–42			
Treatment	BWG (kg)^1^	FI (kg)^2^	FCR _adj._ (kg/kg)^3^	BWG (kg)	FI (kg)	FCR _adj._ (kg/kg)	BWG (kg)	FI (kg)	FCR _adj._ (kg/kg)	Mortality (%)
PC^4^	0.996	1.295^b^	1.300	2.408^a^	3.566^a^	1.481^c^	3.052^a^	4.838^a^	1.586^c^	3.648
NC^5^	0.998	1.317^a^	1.320	2.083^b^	3.337^b^	1.602^a^	2.740^b^	4.670^b^	1.704^a^	5.730
NC + BMD^6^	0.993	1.301^a,b^	1.310	2.100^b^	3.270^b^	1.557^b^	2.716^b^	4.521^b^	1.664^b^	4.167
NC + DFM^7^	0.990	1.292^b^	1.305	2.076^b^	3.253^b^	1.567^b^	2.697^b^	4.501^b^	1.669^b^	4.169
NC + BMD + DFM	1.000	1.301^a,b^	1.307	2.111^b^	3.300^b^	1.563^b^	2.744^b^	4.563^b^	1.664^b^	5.092
SEM	0.015	0.016	0.015	0.049	0.066	0.020	0.084	0.120	0.023	3.219
*P-*value	0.767	0.049	0.181	<0.001	<0.001	<0.001	<0.001	<0.001	<0.001	0.709

Means in the same column followed by different superscript differ significantly (*P* < 0.05, ANOVA, Student–Newman–Keuls test).

^1^BWG—body weight gain.

^2^FI—feed intake.

^3^FCR_adj._—feed conversion ratio adjusted for mortality.

^4^PC—positive control (basal diet without additives or challenge).

^5^NC—negative control (basal diet without additive and challenged birds receiving 3.85 × 10^4^ sporulated oocysts of *Eimeria maxima* + 2.5 × 10^6^ CFU of *Clostridium perfringens*).

^6^BMD—bacitracin methylene disalicylate added in 0.05 g/kg feed.

^7^DFM—direct-fed microbial added 1 g/kg feed (1 × 10^6^ CFU/g of *B. amyloliquefaciens* CECT 5940).

**Table 4.** Effects of supplementation with *Bacillus amyloliquefaciens* CECT 5940 probiotic (DFM), BMD, or its combination on body weight uniformity, carcass, and breast meat yield of challenged broiler at 42 D of age.

Treatment	Body weight uniformity (%)	Carcass yield (%)	Breast meat yield (%)
PC^1^	91.715^a^	77.029^a^	37.453^a^
NC^2^	78.944^b^	75.360^b^	35.640^b^
NC + BMD^3^	88.951^a^	76.271^a^	37.413^a^
NC + DFM^4^	86.295^a^	76.439^a^	37.250^a^
NC + BMD + DFM	84.895^a^	76.763^a^	36.659^a^
SEM	4.737	0.786	0.870
*P-*value	0.001	0.011	0.003

Means in the in the same column followed by different superscript differ significantly (*P* < 0.05, ANOVA, Student–Newman–Keuls test).

^1^PC—positive control (basal diet without additives or challenge).

^2^NC—negative control (basal diet without additive and challenged birds receiving 3.85 × 10^4^ sporulated oocysts of *Eimeria maxima* + 2.5 × 10^6^ CFU of *Clostridium perfringens*).

^3^BMD—bacitracin methylene disalicylate added in 0.05 g/kg feed.

^4^DFM—direct fed microbial added 1 g/kg feed (1 × 10^6^ CFU/g of *B. amyloliquefaciens* CECT 5940).

### Foot Pad Dermatitis and Litter Quality

The effects of experimental treatments on FPD and litter quality scoring are detailed in Table 5. Compared with all the experimental treatments, the incidence of FPD was higher (*P* < 0.05) in challenged broilers of NC group. Irrespective of whether supplemented with DFM or BMD alone or both in association, diets containing the feed additives assessed herein supported similar performance to that exhibited by unchallenged group. At day 28, litter quality scores were lower (*P* < 0.05) in challenged groups receiving DFM or its combination with BMD compared to the challenged group without feed additives or the challenged group receiving only BMD.

**Table 5.** Effects of supplementation with *Bacillus amyloliquefaciens* CECT 5940 probiotic (DFM), BMD, or its combination on Foot pad dermatitis (FPD) and visual litter quality scoring of challenged broiler at 42 D of age.

	FPD	Litter quality visual scoring
Treatment	(from 0 to 4)	(from 1 to 5)
PC^1^	0.250^b^	1.167^b^
NC^2^	1.532^a^	1.594^a^
NC + BMD^3^	0.629^b^	1.667^a^
NC + DFM^4^	0.734^b^	1.179^b^
NC + BMD + DFM	0.584^b^	1.219^b^
SEM	0.447	0.301
*P-*value	<0.001	<0.001

Means in the in the same column followed by different superscript differ significantly (*P* < 0.05, ANOVA, Student–Newman–Keuls test).

^1^PC—positive control (basal diet without additives or challenge).

^2^NC—negative control (basal diet without additive and challenged birds receiving 3.85 × 10^4^ sporulated oocysts of *Eimeria maxima* + 2.5 × 10^6^ CFU of *Clostridium perfringens*).

^3^BMD—bacitracin methylene disalicylate added in 0.05 g/kg feed.

^4^DFM—direct-fed microbial added 1 g/kg feed (1 × 10^6^ CFU/g of *B. amyloliquefaciens* CECT 5940).

### Clostridium perfringens Enumeration by Real-Time PCR Assay

In Table 6, the results for *C. perfringens* enumeration are presented. *Clostridium perfringens* measured in ileal content at days 21 and 28 (1 and 8 D post-infection, respectively) were significantly lower in the groups receiving DFM, BMD, or its combination as compared to the NC group without feed additives (*P* < 0.05) and the values were similar to those of the PC group (*P* > 0.05). There were no differences for *C. perfringens* enumeration in the cecal content at days 21 (*P* = 0.060) or 28 (*P* = 0.076), but only tendency to be lower in the treatment groups compared to NC group.

**Table 6.** Effects of supplementation with *Bacillus amyloliquefaciens* CECT 5940 probiotic (DFM), bacitracin methylene disalicylate (BMD) or its combination on *Clostridium perfringens* enumeration (*plc* gene copies/g digesta) in cecal and ileal contents of broilers.

	Ileum		Cecum	
	(log_10_ gene copies/g digesta)		(log_10_ gene copies/g digesta)	
Treatment	21 D	28 D	21 D	28 D
PC^1^	5.36^b^	5.25^b^	5.84	5.71
NC^2^	5.85^a^	5.70^a^	6.11	5.92
NC + BMD^3^	5.07^b^	4.82^b^	5.49	5.35
NC + DFM^4^	5.24^b^	4.99^b^	5.68	5.43
NC + BMD + DFM	5.17^b^	4.90^b^	5.53	5.40
SEM	0.420	0.355	0.481	0.487
*P-*value	0.003	<0.001	0.060	0.076

Means in the in the same column followed by different superscript differ significantly (*P* < 0.05, ANOVA, Student–Newman–Keuls test).

^1^PC—positive control (basal diet without additives or challenge).

^2^NC—negative control (basal diet without additive and challenged birds receiving 3.85 × 10^4^ sporulated oocysts of *Eimeria maxima* + 2.5 × 10^6^ CFU of *Clostridium perfringens*).

^3^BMD—bacitracin methylene disalicylate added in 0.05 g/kg feed.

^4^DFM—direct-fed microbial added 1 g/kg feed (1 × 10^6^ CFU/g of *B. amyloliquefaciens* CECT 5940).

### Gut Health

The parameters evaluated at 21 and 28 D for intestinal health and their frequency of occurrence are presented in Table 7. The challenge significantly increased the abnormal content in the challenged group compared to the PC group (*P* < 0.05). In addition, the other gut health traits assessed (thin or fragile intestinal walls, ballooning in the gut, sloughed mucosa and swollen or red serosal and mucosal surface) were not significantly different between treatments (*P* > 0.05). Both lack in muscular tone or presence of undigested feed in the colo-rectum segment were not observed in the birds during the evaluation.

**Table 7.** Frequency^1^ of intestinal health evaluation measurements of broilers under challenge conditions fed diets supplemented with *Bacillus amyloliquefaciens* CECT 5940 probiotic (DFM), BMD its combination at 21 and 28 D of age.

	Abnormal content		Thin or fragile intestinal walls		Ballooning in the gut		Sloughed mucosa		Swollen or red (serosal/mucosal surface)	
Treatment	21 D	28 D	21 D	28 D	21 D	28 D	21 D	28 D	21 D	28 D
PC^2^	1 (1%)^b^	11 (9%)^a,b^	3 (2%)	1 (1%)	3 (2%)	6 (5%)^b^	11 (9%)	9 (7%)	3 (2%)	11 (9%)^a,b^
NC^3^	15 (12%)^a^	18 (14%)^a^	2 (2%)	3 (2%)	7 (6%)	16 (13%)^a^	9 (7%)	15 (12%)	2 (2%)	16 (13%)^a^
NC + BMD^4^	13 (10%)^a^	6 (5%)^b^	2 (2%)	5 (4%)	7 (6%)	10 (8%)^a,b^	11 (9%)	12 (10%)	4 (3%)	6 (5%)^b^
NC + DFM^5^	16 (13%)^a^	7 (6%)^b^	1 (1%)	6 (5%)	8 (6%)	6 (5%)^b^	8 (6%)	13 (10%)	4 (3%)	6 (5%)^b^
NC + BMD + DFM	16 (13%)^a^	7 (6%)^b^	3 (2%)	6 (5%)	7 (6%)	6 (5%)^b^	8 (6%)	10 (8%)	3 (2%)	6 (5%)^b^
*P-*value	<0.0001	<0.0001	0.845	0.217	0.464	0.0012	0.7395	0.2368	0.906	0.0008

Values in the in the same column with different superscripted letters differ significantly (*P* < 0.05) by the non-parametric Kruskal–Wallis test followed by Dunn-Bonferroni test.

^1^Number of positive observations are presented followed by the frequency in parenthesis.

^2^PC—positive control (basal diet without additives or challenge).

^3^NC—negative control (basal diet without additive and challenged birds receiving 3.85 × 10^4^ sporulated oocysts of *Eimeria maxima* + 2.5 × 10^6^ CFU of *Clostridium perfringens*).

^4^BMD—bacitracin methylene disalicylate added in 0.05 g/kg feed.

^5^DFM—direct-fed microbial added 1 g/kg feed (1 × 10^6^ CFU/g of *B. amyloliquefaciens* CECT 5940).

The frequencies of thin intestinal wall and sloughed mucosa were not significantly different between dietary treatments (*P* > 0.05) at 28 D of age. The frequencies observed for abnormal content and swollen gut mucosa in the NC group without feed additives were higher compared to the groups receiving DFM, BMD, or its combination (*P* < 0.05), but was not different from the PC group (*P* > 0.05). On the other hand, the observed frequencies for ballooning in the challenged group receiving only BMD was not significantly different from the PC group without feed additives (*P* > 0.05).

## DISCUSSION

The present study evaluated the performance of broilers fed DFM, BMD, and its combination under an enteric pathogen challenge. Although we have used the challenge model with *Eimeira* and *C. perfringens*, our evaluation was aimed at understanding the framework of disturbances in the microbiota that occur in a model close to the subclinical condition of necrotic enteritis. The whole effects addressed below, which took into account challenged broilers, must be understood as the sum of effects caused by the inoculation of both microorganisms, since the control groups used for comparation do not include challenged broilers with neither *Eimeria* nor *Clostridium* separately. The treatment control groups used here are widely described in the literature in trial conducted aiming induction of necrotic enteritis (Qing et al., 2017; Wu et al., 2018; Hofacre et al., 2019).

The results present in Table 3 support the hypothesis that *B. amyloliquefaciens CECT* 5940 can provide similar performance in broilers fed diets containing AGP, here represented by BMD. Up to day 21, there was no significant effect of the challenge models used, but on days 35 and 42, the drop in the performance was evident compared to the PC group. The poor weight and feed efficiency usually occur from 4 to 9 D post-inoculation with *Eimeria*.

Although BWG and FI were not significantly improved with the supplementation of DFM, BMD or its combination on days 35 and 42 compared to NC group without feed additives, there was an improvement in FCR. Jayaraman et al. (2013), inducing broilers to necrotic enteritis disease and providing supplemented diets with DFM based on Bacillus spp., did not observe differences in BWG at 35 D, but noticed a better FCR. However, broilers fed diets supplemented with feed additives did not have similar performance as observed in the PC group. The same outcomes were observed by Wang et al. (2017) in which broilers of the NC group had the same FCR as observed in broilers challenged and fed diet with probiotic, both being worse when compared to those broilers of PC group.

The reason behind this is that the applied challenge model might have been strong enough to partially prevent the total recovery of the birds in such time, mainly characterized by the big drop in FI. Generally, a compensatory growth can be observed after the challenge period (Arczewska-Włosek and Świątkiewicz, 2013). However, no improvement in BWG at 35 or 42 D among challenged broilers was observed. Thus, it was not substantial in the study proposed herein.

It is interesting to observe that the better FCR indicated that the birds were more efficient to use the nutrients for muscle deposition and this might have resulted in the significant better uniformity, carcass, and breast meat yields observed in the groups receiving DFM, BMD, or its combination and these results were comparable to the PC group (Table 4). It is also reasonable to consider that different enzyme compounds are produced by *Bacillus amyloliquefaciens* CECT 5940 such as cellulase, protease, amylase, and xylanase (Diaz, 2007). Thus, the inclusion of *B. amyloliquefaciens* may have a positive effect on nutrient digestibility and absorption, due to the presence of exogenous enzymes in the intestinal lumen (Farhat-Khemakhem et al., 2017).

The results presented in Table 5 indicate that supplementation with *Bacillus amyloliquefaciens* CECT 5940 alone or in combination with BMD provided lower wet litter scores as observed in the PC group. Consequently, the supplementation of *Bacillus amyloliquefaciens* CECT 5940 also reduced the footpad dermatitis scores. Our outcomes are in line with those of Whelan et al. (2018) which report that broilers fed diet supplemented with *Bacillus subtilis* or AGP had lower mean score of footpad dermatitis in comparison to broilers of NC group. Although there was a difference in strains used in our trial compared with the author aforementioned, we believe that the similarity on results are due the mechanism of action most commonly shared by the most of *Bacillus spp.* which refers to the competitive exclusion. Through this mechanism, the population of *C. perfringens* has diminished in the ileum of broilers in both trials helping to keep gut integrity. This might have contributed for better nutrient utilization and is frequently related to better litter quality (Lei et al., 2015). Furthermore, poor quality of litter can increase incidence of FPD, which in turn are observed in necrotic enteritis (Timbermont et al., 2011).

The supplementation of the diets with DFM, BMD, or its combination was also effective in reducing *C. perfringens* counts observed in the ileum of the birds on days 21 and 28 (Table 6). A trend (*P* < 0.10) for reduction in *C. perfringens* was also observed in cecum on days 21 and 28; however, the high diversity of the cecal microbiota may contribute for a more stable environment (Bjerrum et al., 2006; Pourabedin and Zhao, 2015; Blajman et al., 2017; De Cesare et al., 2017). The mechanisms of inhibition of antibiotics are well known, but *Bacillus amyloliquefaciens* CECT 5940 have the different mode of action to inhibit the action of *C. perfringens* by modulating quorum sensing system, i.e., by interrupting communication between these pathogenic bacteria (Ortiz et al., 2016). Other mechanisms may involve the production of secondary metabolites such as lactic acid (Diaz, 2007) and bacteriocins (Mantovani et al., 2011).

The effect of the challenge on performance was not evident until 21 D, but the necropsy done in this age indicated an increase in the presence of abnormal content compared to the PC group (Table 7). According to Teirlynck et al. (2011), abnormal content is characterized by excess of mucus with presence of some blood and gases which indicates an initial appearance of dysbiosis. After 7 D, the results observed on day 28 showed that the supplementation of *Bacillus amyloliquefaciens* CECT 5940 in combination or not with BMD significantly decreased the frequency of abnormal content and the swollen mucosa compared to the NC group without additives. Either swelling or redness is common and classical signs of inflammation process due to toxins produced by *C. perfringens* (Guo et al., 2015). The supplementation of DFM alone or combined with BMD, but not DFM alone, significantly reduced the frequency of ballooning compared to the NC group without additives. Ballooning is a classical consequence of dysbiosis, being characterized by the visible enlargement in gut diameter and the presence of liquid, slimy, or gases (Pattison, 2002; De Gussem, 2007). Previous findings also found that broilers challenged with *Eimeria* species and *C. perfringens* exhibited an increase in ballooning in the gut (Jayaraman et al., 2013). Therefore, the reduction in frequencies of abnormal content, ballooning, and inflammation could be related to the decrease in the population of *C. perfringens* with the supplementation of *B. amyloliquefaciens* CECT 5940 as observed in Table 6.

In conclusion, dietary supplementation of *B. amyloliquefaciens* CECT 5940 can totally or partially replace AGPs in the diets of broiler chickens due to its beneficial effects in improving FCR, reducing CP in ileum, improved uniformity, carcass and breast meat yield during an enteric pathogen challenge. Therefore, these improvements on the performance may be attributable to better intestinal health and litter quality.
